# Effects and safety of extracorporeal membrane oxygenation in the treatment of patients with ST-segment elevation myocardial infarction and cardiogenic shock: A systematic review and meta-analysis

**DOI:** 10.3389/fcvm.2022.963002

**Published:** 2022-09-27

**Authors:** Shuo Pang, Guangrui Miao, Xiaoyan Zhao

**Affiliations:** Department of Cardiology, The First Affiliated Hospital of Zhengzhou University, Zhengzhou, China

**Keywords:** ECMO, ST-segment elevated myocardial infarction, mortality, cardiogenic shock, complications, prognosis

## Abstract

**Background:**

There is a lack of large randomized controlled trials (RCTs) that comprehensively evaluate the effects of venoarterial extracorporeal membrane oxygenation (V-A ECMO)- assisted treatment of patients with ST-segment elevation myocardial infarction (STEMI) combined with Cardiogenic shock (CS). This meta-analysis aims to identify predictors of short-term mortality, and the incidence of various complications in patients with STEMI and CS treated with V-A ECMO.

**Methods:**

We searched PubMed, Cochrane Library, Web of Science, Embase, China National Knowledge Infrastructure (CNKI), and the Wanfang Database from 2008 to January 2022 for studies evaluating patients with STEMI and CS treated with V-A ECMO. Studies that reported on mortality in ≥ 10 adult (>18 years) patients were included. Newcastle-Ottawa Scale was used by two independent reviewers to assess methodological quality. Mantel-Haenszel models were used to pool the data for meta-analysis.

**Results:**

Sixteen studies (1,162 patients) were included with a pooled mortality estimate of 50.9%. Age > 65 years, BMI > 25 kg/m^2^, lactate > 8 mmol/L, anterior wall infarction, longer CPR time, and longer time from arrest to extracorporeal cardiopulmonary resuscitation (ECPR) were risk predictors of mortality. Achieving TIMI-3 flow after percutaneous coronary intervention (PCI) was a protective factor of mortality. The prevalence of bleeding, cerebral infarction, leg ischemia, and renal failure were 22, 9.9, 7.4, and 49.4%, respectively.

**Conclusion:**

Our study identified Age, BMI, lactate, anterior wall infarction, TIMI-3 flow after PCI, CPR time, and time from arrest to ECPR significantly influence mortality in STEMI patients with CS requiring V-A ECMO. These factors may help clinicians to detect patients with poor prognoses earlier and develop new mortality prediction models.

## Introduction

In recent years, the incidence of acute ST-segment elevation myocardial infarction (STEMI) has been increasing year by year with a trend toward younger patients (1). Cardiogenic shock (CS) is the leading cause of death in patients with myocardial infarction (MI) and occurs in approximately 8–10% of STEMI patients (2). Despite improvements in therapeutic agents, shorter door-to-balloon (D2B) time, and optimized reperfusion strategies, mortality in patients with STEMI combined with CS remains as high as 50% (3). Even after discharge, patients with STEMI combined with CS are often left with a variety of serious complications, leading to repeated hospitalizations and poor long-term prognosis (4, 5). To overcome the limitations of vasopressors, maintain adequate perfusion pressure, and prevent multi-organ failure, mechanical circulatory support (MCS) is attractive for improving hemodynamics and clinical outcomes in patients with STEMI combined with CS (6). Venoarterial extracorporeal membrane oxygenation (V-A ECMO) is a cardiopulmonary assist device, which not only oxygenates the blood but also replaces the heart to provide power for intravascular circulation, playing the role of partial or total replacement of the heart. It has been widely used in the resuscitation treatment of patients with STEMI combined with CS (7).

However, there is a lack of large RCTs that comprehensively evaluate the effects of V-A ECMO-assisted treatment of patients with STEMI combined with CS and there is a lack of well-recognized predictive scores for the assessment of patient prognosis. SAVE score (8) is the ECMO score for CS, which can be applied to all cardiovascular diseases. It is not specific to myocardial infarction, and patients with extracorporeal cardiopulmonary resuscitation (ECPR) are excluded. In addition, the SAVE score is complex and not suitable for emergent evaluation. Encourage score (9) and AMI-ECMO (10) score have emerged to assess the clinical benefit of V-A ECMO used in patients with AMI. However, the predictive ability is limited due to the fact that data is from small-scale studies, which may ignore some important risk predictors. Complications such as bleeding, vascular complications, lower limb ischemia, renal impairment, infection, and myocardial stunning are often present during V-A ECMO support, but data from large-scale studies in STEMI patients is still limited (11). How to select patients with STEMI and CS who are most likely to benefit from V-A ECMO support while avoiding excessive waste of medical resources has become a very important decision in clinical practice.

To address this knowledge gap, we conducted a systematic review and meta-analysis to evaluate the predictors of short-term mortality which was defined by 30-day and in-hospital mortality and the incidence of various complications in patients with STEMI and CS treated with V-A ECMO. The clinical evidence summarized in this study could help clinicians to select the best population for V-A ECMO supported treatment, improve awareness of complications treatment.

## Methods

The protocol for this systematic review was registered on the PROSPERO database^1^ and conducted following the Preferred Reporting Items for Systematic Reviews and Meta-Analyses guidelines (12).

### Data sources and searches

We systematically searched and identified relevant studies from six databases, including PubMed, Cochrane Library, Web of Science, Embase, China National Knowledge Infrastructure (CNKI), and the Wanfang Database from 2008 to January 2022. Controlled vocabulary supplemented with keywords was used to search for patients with STEMI and CS treated with V-A ECMO. The detailed search strategy is presented in the Supplementary material.

### Study selection, inclusion, and exclusion criteria

We performed an initial screening based on the titles and abstracts. If uncertain, full-text studies were selected for careful reading and analysis against the eligibility criteria (inclusion/exclusion). Studies that reported on mortality in ≥ 10 adults (>18 years) patients with STEMI and CS necessitating V-A ECMO were included. Studies designed as case reports, reviews, animal studies, studies without data specifically for STEMI patients, and studies without relevant outcomes were excluded. If two studies report on the same cohort or sampling from the same population, we included the study with the larger population or both studies only if they reported on different predictors.

### Evaluation of study quality

The Newcastle–Ottawa scale (NOS) was used for the analysis of the quality of the studies included in this review. Using this scale, each study is categorized into three groups: selection, comparability, and outcome. For the selection and outcome categories, the studies were awarded a star for each item. For the comparability category, two stars were awarded. A study with more than six stars is considered to be of high quality, with a maximum of nine stars (13).

### Data abstraction

Two researchers (Pang and Miao) independently extracted data from all eligible studies. Any differences were discussed or decided by a third reviewer. The following data were extracted from the selected studies: author, publication year, study design, number of studies, follow-up period, 30-day mortality, in-hospital mortality, the incidence of bleeding, the incidence of neurological injury, incidence of lower limb ischemia, and incidence of acute renal insufficiency. We only extracted independent predictors of all-cause mortality from multivariable analysis (estimator, effect estimate, and 95% confidence intervals [CIs]).

### Data synthesis and statistical analysis

Heterogeneity among studies was estimated using the chi-square test-based I^2^ statistic. I^2^ Values of <50, 50–75, and >75% were classified as low, medium, and high heterogeneity, respectively. A Mantel-Haenszel model was used to calculate pooled risk ratios (RR) and 95% CI. If heterogeneity was low (*P* > 0.10, I^2^ < 50%), a fixed-effect model was used to calculate the combined RR values. Otherwise, a random-effect model was used. Publication bias was estimated graphically using a funnel plot. An asymmetric funnel plot may indicate a possible publication bias. To evaluate predictors of mortality when using V-A ECMO, we used the inverse variance method to report a pooled overall estimate with its respective 95 percent confidence intervals (95% CIs). In addition, Sensitivity analysis was performed *via* the leave-one-out method to assess the influence of individual studies on the pooled estimate. A pooled effect estimate was calculated if the predictor was identified in more than one study using a similar or roughly similar definition. The data were analyzed using R software version 4.1.2 statistical software. A value of *p* < 0.05 was considered statistically significant.

## Results

**FIGURE 1:**
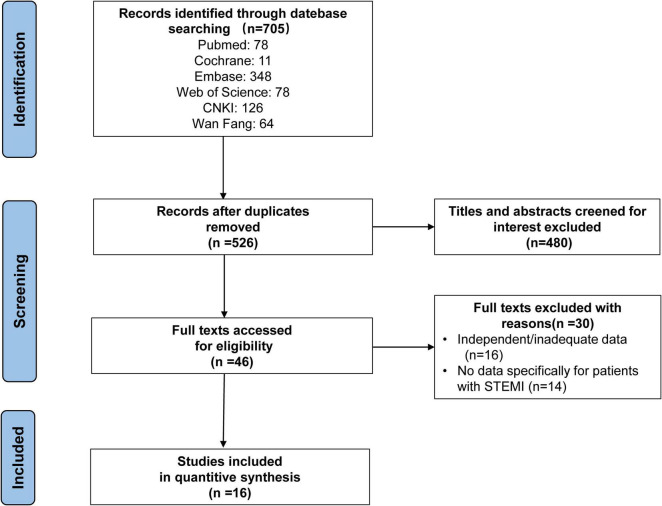
Literature search strategy.

The search strategy identified 705 abstracts, of which 16 studies (10, 14–28), representing 1,162 patients met the inclusion criteria. Fifteen studies were retrospective cohort studies and one study was a randomized controlled trial (RCT) study. The detailed retrieval process is shown in Figure 1.

As shown in Table 1, according to the NOS, twelve studies with scores higher than six stars were considered high quality, and the remaining three studies were medium quality for five and six stars, respectively. The characteristics of the selected studies are presented in Table 2.

**TABLE 1:** Newcastle-Ottawa scale.

References	Selection				Compar ability	Outcome			
			
	Representa tiveness of the exposed cohort	Selection of the non-exposed cohort	Ascertain ment of exposure	Demons tration that outcome of interest was not present at start of study	Compar ability of cohorts on the basis of the design or analysis	Assess ment of outcome	Was follow-up long enough for outcomes to occur	Adequacy of follow up of cohorts	Total scores
Chung et al. (14)	★	★	★	★	★	★	★	★	8
Lee et al. (15)	★	★	★	★	★	★	★		7
Semaan et al. (16)	★	★	★	★	★	★	★	★	8
Szczanowicz et al. (17)	★	★	★	★	★	★	★		7
Wu et al. (18)	★	★	★	★	★	★	★		7
Li et al. (19)	★	★	★	★	★	★	★		7
Cho et al. (20)	★	★	★	★	★★	★	★		8
Liu et al. (21)	★	★	★	★	★	★	★		7
Liang et al. (22)	★	★	★	★	★★	★	★		8
Sheu et al. (23)	★		★	★	★	★			5
Huang et al. (24)	★	★	★	★	★	★	★	★	8
Choi et al. (10)	★	★	★	★	★★	★	★		8
Pahuja et al. (25)	★		★	★	★	★			5
Fu et al. (26)	★	★	★	★	★	★	★		7
van den Brink et al. (27)	★		★	★	★	★	★		6

**TABLE 2:** Characteristics of included studies.

References	Year	Country	Patients (*n*)	Male (*n*)	Cardiogenic shock definition	Mortality
Chung et al. (14)	2016	Taiwan, China	65	58	SBP < 90 mmHg, pulmonary edema, low cardiac output	30-day
Lee et al. (15)	2016	Taiwan, China	51	45	SBP < 90 mm Hg for > 30 min, high-dose inotropes, IABP,	30-day
Semaan et al. (16)	2021	France	51	41	Refractory cardiogenic shock	30-day
Szczanowicz et al. (17)	2021	Germany	79	65	Hypotension, high-dose inotropes	30-day
Wu et al. (18)	2018	China	37	29	STEMI with cardiac arrest	In-hospital
Li et al. (19)	2021	China	28	24	STEMI with ECMO and PCI, cardiac arrest	In-hospital
Cho et al. (20)	2018	Korea	42	28	SBP < 90 mm Hg for > 30 min, high-dose inotropes, cardiac arrest/CPR	30-day
Liu et al. (21)	2015	China	19	16	Cardiac shock, cardiac arrest/CPR	In-hospital
Liang et al. (22)	2021	China	43	30	STEMI with cardiac arrest	In-hospital
Sheu et al. (23)	2010	Taiwan, China	46	NA	SBP < 90 mm Hg for > 30 min, low cardiac output, high-dose inotropes	30-day
Huang et al. (24)	2018	Taiwan, China	46	40	Killip IV, SBP < 90 mm Hg, high-dose inotropes, cardiac arrest/CPR	30-day
Choi et al. (10)	2018	Korea	145	110	Refractory CS, cardiac arrest	30-day
Pahuja et al. (25)	2020	US	444	NA	NA	In-hospital
Fu et al. (26)	2017	China	27	21	Cardiac arrest	In-hospital
van den Brink et al. (27)	2021	Netherland	18	15	Killip IV	In-hospital
Brunner et al. (28)	2019	Germany	42	NA	Cardiac shock	30-day

### Mortality

**FIGURE 2:**
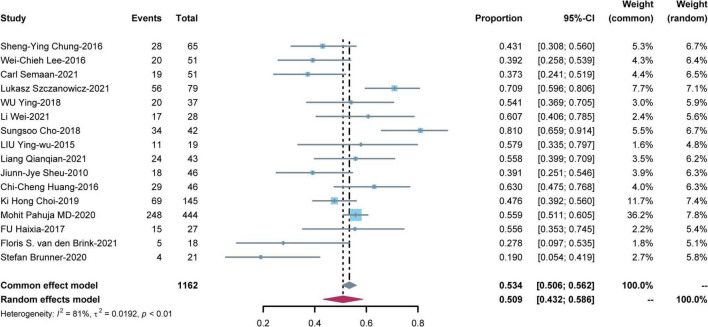
Meta-analysis of short-term mortality for patients on venoarterial extracorporeal membrane oxygenation (V-A ECMO) for cardiogenic shock (CS) complicating ST-segment elevation myocardial infarction (STEMI).

Sixteen studies reported short-term mortality for patients on V-A ECMO for CS complicating STEMI (Figure 2). The pooled estimate of the OR was 0.509 [95% CI: (0.432, 0.586)] in a random effects model with significant heterogeneity (I^2^ = 81%, *P* < 0.01). In sensitivity analysis, the overall effect size was not substantially affected after removing each study in turn (Supplementary Figure 1).

### Predictors

**FIGURE 3:**
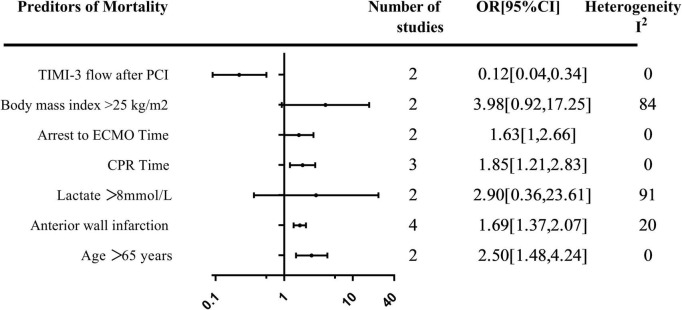
Summary of meta-analysis of predictors of mortality.

A total of seven predictors of mortality were evaluated in more than one study. The summary of the pooled effect of the predictors that were meta-analyzed was provided in Figure 3.

### Demographics predictors

The effect of age > 65 years on mortality was evaluated in two studies. Age greater than 65 years was associated with an increase in mortality [OR, 2.50; 95% CI: (1.48, 4.24)] (Supplementary Figure 2). The relationship between BMI > 25 kg/m^2^ and mortality was evaluated in two studies with a total of 196 patients, all using multi-variable analysis. Pooled estimates showed a trend toward increased mortality in patients whose BMI > 25 kg/m^2^ [OR, 3.98; 95% CI: (0.92, 17.25)] (Supplementary Figure 3).

### Biochemical and angiographic predictors

The relationship between lactate > 8 mmol/L prior to V-A ECMO initiation and mortality was evaluated in two studies with 224 patients [OR, 2.90; 95% CI: (0.36, 23.61)] (Supplementary Figure 4). Achieving TIMI-3 flow after percutaneous coronary intervention (PCI) was reported in two studies with 361 patients. A pooled estimate of TIMI-3 flow after PCI was associated with decreased mortality [OR, 0.12; 95% CI: (0.04, 0.34)] (Supplementary Figure 5). The effect of anterior wall infarction on mortality was evaluated in four studies with 135 patients. The pooled estimate demonstrated a 1.69-fold increase in mortality in patients with anterior wall infarction [OR, 1.69; 95% CI: (1.37, 2.07)] (Supplementary Figure 6).

### Arrest predictors

The effect of cardiopulmonary resuscitation (CPR) time on mortality was evaluated in three studies with 112 patients. Longer CPR duration was associated with an increase in mortality [OR, 1.85; 95% CI: (1.21, 2.83)] (Supplementary Figure 7). Time from arrest to V-A ECMO cardiopulmonary resuscitation (ECPR) time was assessed in two studies with 70 patients with a pooled effect estimate revealing a 1.63-fold increase in mortality in patients with longer time [OR, 1.63; 95% CI: (1, 2.66)] (Supplementary Figure 8).

### Complications

In our study, complications including bleeding, leg ischemia, cerebral infarction, and renal failure occurred during hospitalization.

#### Bleeding

**FIGURE 4:**
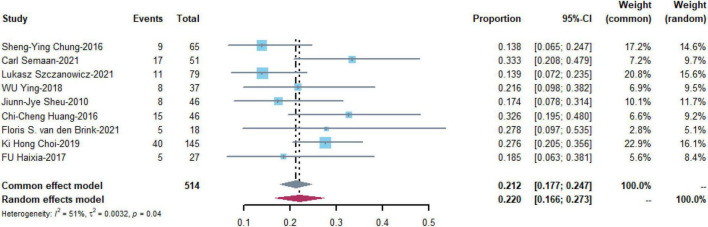
Meta-analysis of the prevalence of bleeding.

Bleeding was the most commonly reported complication and was reported in nine studies with 514 patients. The pooled estimate of the OR was 0.220 [95% CI: (0.166, 0.273)] in a random effects model with medium heterogeneity (I^2^ = 51%, *P* = 0.04) (Figure 4).

#### Cerebral infarction

**FIGURE 5:**
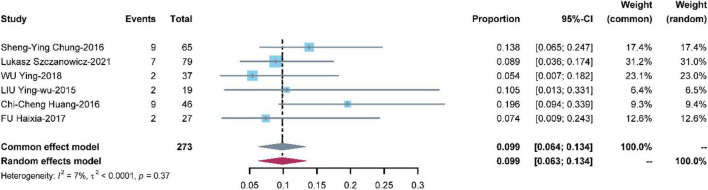
Meta-analysis of the prevalence of cerebral infarction.

Seven studies with 273 patients reported cerebral infarction. The pooled estimate of the OR was 0.099 [95% CI: (0.064, 0.134)] in a common effects model with low heterogeneity (I^2^ = 7%, *P* = 0.37) (Figure 5).

### Leg ischemia

**FIGURE 6:**
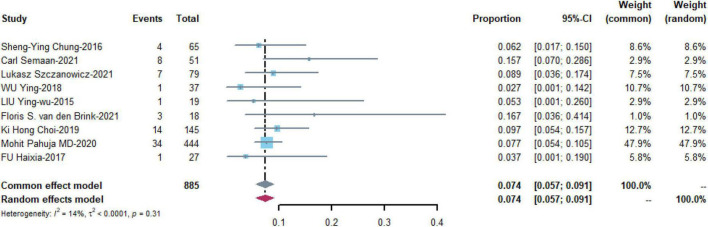
Meta-analysis of the prevalence of leg ischemia.

Nine studies with 885 patients assessed the prevalence of leg ischemia with a pooled estimate of 0.074 [95% CI: (0.057, 0.091)] and low heterogeneity among the studies (I^2^ = 14%, *p* = 0.31) (Figure 6).

#### Renal failure

**FIGURE 7:**
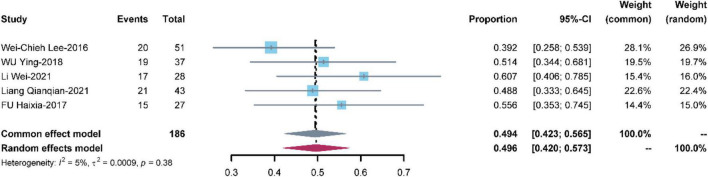
Meta-analysis of the prevalence of renal failure.

Five studies with 186 patients reported renal failure. The pooled renal failure rate was 0.494 [95% CI: (0.423, 0.565)] with low heterogeneity (I^2^ = 5%, *P* = 0.38) (Figure 7).

### Risk of bias assessment

**FIGURE 8:**
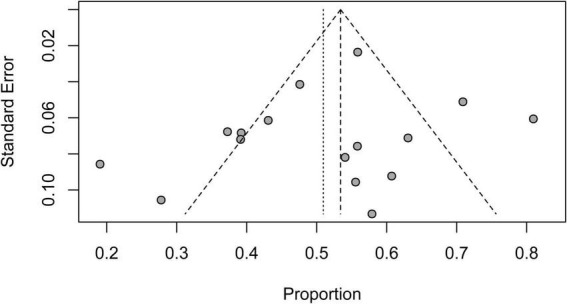
Funnel plot for the studies reporting short-term mortality.

The funnel plot was asymmetric when short-term mortality for patients on V-A ECMO for CS complicating STEMI was analyzed, which indicated potential publication bias. Six studies contributed to the asymmetry as they fell outside the 95% CI funnel (Figure 8).

## Discussion

Patients with severe STEMI have larger myocardial infarct size, often presenting with CS, and the persistent hypoperfusion state may lead to multiple organ failure. As a short-term extracorporeal life support device, V-A ECMO has become the first-line treatment for CS because of its convenience, effectiveness, and the advantage of providing both circulation and respiratory support (23). Although there have been some retrospective studies on patients with STEMI and CS supported by V-A ECMO, large RCTs and well-recognized predictive scores are lacking for assessing patient outcomes.

The short-term mortality of STEMI and CS patients treated with V-A ECMO was estimated to be as high as 50.9% in this meta-analysis of 16 studies involving 1,162 patients. Age > 65 years, BMI > 25 kg/m^2^, lactate > 8 mmol/L, anterior wall infarction, longer CPR time, and longer time from arrest to ECPR were independent risk predictors of mortality. Achieving TIMI-3 flow after PCI was a protective factor of mortality. The prevalence of bleeding, cerebral infarction, leg ischemia, and renal failure were 22, 9.9, 7.4, and 49.4%, respectively.

The short-term mortality rate of patients with STEMI and CS after receiving V-A ECMO was 50.9% in our study. However, the only RCT study on V-A ECMO use in myocardial infarction patients with CS showed a 30-day mortality rate of 19% in patients receiving V-A ECMO support and 33% in non-V-A ECMO-supported patients (28). The mortality rate in the RCT study was lower than in the present study, and the large difference may be explained by the fact that those who were selected for the RCT study had timely revascularization, optimal vasoactive drug administration, and were excluded from higher-risk groups (all under 80 years old, shock duration < 12 h, etc.). Data from the Extracorporeal Life Support Organization (ELSO) showed that V-A ECMO-assisted myocardial infarction patients had a survival rate of 39.5% (8), whereas the relatively high survival rate in this study may be attributed to improvements in treatment strategies and therapeutic agents in recent years. The heterogeneity remained high across studies. This may be because most of the included studies were retrospective studies with small sample sizes from different hospitals. In addition, the success proportion of PCI, and the duration of cardiac arrest may also have influenced the mortality. Therefore, high-quality RCTs are urgently needed to resolve this issue.

This meta-analysis summarizes the same predictors of mortality reported in multivariable regression models in different studies in order to provide a reference for developing new clinical prediction models. Advanced age is a common risk factor for MI, and our meta-analysis showed that age > 65 years increases short-term mortality in patients with STEMI with CS assisted by V-A ECMO. Previous studies have shown that age > 75 years is a relative contraindication to ventricular mechanical assist device implantation. When we analyzed the literature, we found that some older people can still benefit greatly from the use of V-A ECMO. Therefore, older patients with favorable prognostic factors should be carefully selected (29). Lactate serves as a valuable biomarker, and its increase correlates with the degree of tissue hypoxia. It is also a biomarker that is relatively easy to measure at the bedside due to the availability of handheld lactate meters and the satisfactory agreement between peripheral venous, central venous, and arterial lactate values (30). Lactate has been widely used to predict the prognosis of patients with different types of shock. A great many studies have shown lactate and lactate clearance are strongly associated with prognosis in patients with CS treated with V-A ECMO (31). Obesity is a well-recognized risk factor for poor prognosis and mortality in patients with cardiovascular disease, similar to the pooled results of this meta-analysis (32, 33). However, in the field of critical medicine, sometimes BMI and death are not positively correlated, which is known as the “obesity paradox,” which suggests that when patients gain weight, they have a better nutritional supply and resistance to disease (34). Previous studies have shown a significant increase in mortality in patients receiving V-A ECMO when BMI was < 18.5 kg/m^2^ (35). Whereas, Sreenivasan et al. (36) reported that when patients with acute heart failure were assisted with V-A ECMO, there was no significant association between BMI and mortality, while patients treated with V-A ECMO for AMI had a BMI > 35 kg/m^2^ and mortality was strongly associated. Older age, higher BMI, and higher lactate were significant predictors of mortality in different prediction models of V-A ECMO-supported AMI and CS patients (encouragement score, SAVE score, and AMI-ECMO score). Encourage score and SAVE score are the most commonly used prediction models, but they do not include angiographic data such as the location of the culprit vessel and whether or not achieving TIMI III after PCI, which are also important factors influencing the prognosis of myocardial infarction (9). AMI-ECMO score is the only risk score including angiographic data and has shown a better predictive effect than Encourage score and SAVE score, but it still needs to be confirmed by multicenter studies and more patients enrolled (8–10).

In addition to the common predictors of mortality mentioned above, this meta-analysis also found that CPR time and time from arrest to ECPR were also independent predictors of death. Prolonged CPR is associated with poor outcomes, with only a few patients able to return to their previous lives without complications. Previous studies have shown that standard CPR can provide up to 25–40% of normal cardiac output and CPR time longer than 40 min is strongly associated with poor prognosis (37). The time from arrest to ECPR may be a major determinant of a good outcome (38). When ECPR is provided rapidly, improved oxygenation to vital organs may prevent organ failure. This is particularly true for myocardial viability, as ECPR improves coronary oxygenation, which may affect the recovery of spontaneous circulation. Park et al. (39) reported that time from arrest to ECPR was significantly and inversely associated with survival to discharge for every 10 min increase in time and time from arrest to ECPR ≤ 60 min was independently associated with improved survival. Therefore, for patient prognosis, it is extremely important to shorten the time of hypoperfusion before V-A ECMO treatment and guarantee blood pressure stability after V-A ECMO implantation by optimizing the standardized CPR process and deciding early ECMO support (40). However, for STEMI patients, the implementation of V-A ECMO before PCI remains controversial. Longer D2B time is closely associated with higher mortality, even if they are treated within 90 min of admission (41). Huang et al. (24) reported that although D2B time may be prolonged, the benefits of early ECMO support may compensate for the damage. However, the result should be interpreted with caution, as no large sample studies have confirmed this, and future studies are needed to confirm this.

Venoarterial extracorporeal membrane oxygenation-assisted therapy requires a balance of benefit and risk of complications. Despite advances in material and technical improvements, bleeding and thromboembolism remain major threats to V-A ECMO therapy (42). As an invasive ventricular assist device, bleeding complications often occur during the use of V-A ECMO, due to the continuous application of anticoagulants and consumption of clotting substances in the process of diversion (43). In this study, the incidence of bleeding complications was 22.0%, suggesting that in the application of V-A ECMO, clotting function should be closely monitored, the dosage of heparin should be adjusted in time and the occurrence of bleeding complications should be detected early. Ischemic stroke occurred in 9.9% of patients during V-A ECMO-assisted therapy, and the causes of stroke were multifactorial: thrombosis, anticoagulation, hemodynamic instability, etc. Compared with patients without neurological complications, mortality was significantly higher in patients with stroke (44). With prolonged bed rest and large caliber femoral artery cannulation, patients often suffer from ischemic complications during V-A ECMO treatment. Lamb et al. (45) reported that none of the 55 patients with prophylactic distal perfusion catheters had limb ischemia, while 12 of the 36 patients without distal perfusion catheters had limb ischemia. Juo et al.’s (46) meta-analysis also reported a large benefit from the use of a distal perfusion catheter, which is therefore strongly recommended. Our meta-analysis showed that the incidence of renal failure remains as high as 49.4%. STEMI patients with CS are prone to acute renal insufficiency. Previous studies have shown that high volume load status is closely related to patient mortality, so CRRT therapy should be performed as soon as possible to reduce volume load and promote recovery (47).

There is a lack of large RCTs and well-recognized predictive scores to assess the outcomes of STEMI patients with CS requiring V-A ECMO. To overcome the shortcomings of the previous studies, our study not only identified some factors that significantly influence mortality in STEMI patients with CS treated with V-A ECMO but also further discussed the clinical application of these risk factors according to the latest clinical progress. These factors may help clinicians develop new mortality prediction models and select patients who are most likely to benefit from V-A ECMO support by balancing the benefits and risks.

## Limitation

Our study has some limitations. The results from this meta-analysis must be interpreted with caution. First, most of the included studies were non-RCT studies, which increased the risk of bias. There was high heterogeneity among the included studies, but we have conducted sensitivity analysis to explore the potential sources of heterogeneity. Subgroup analyses were also attempted but no meaningful group assignments were identified. Second, we pooled together different definitions of short-term mortality, which may imply some variation in the results of different studies. Third, the number of included studies was small. Finally, this study assessed only short-term mortality and provided limited insight into long-term survival and recovery of cardiac function.

## Conclusion

Our study identified Age, BMI, lactate, anterior wall infarction, TIMI-3 flow after PCI, CPR time, and time from arrest to ECPR significantly influence mortality in STEMI patients with CS requiring V-A ECMO. These factors may help clinicians to detect patients with poor prognosis earlier and develop new mortality prediction models.

## Data availability statement

The original contributions presented in this study are included in the article/Supplementary material, further inquiries can be directed to the corresponding author.

## Author contributions

SP and XZ: conceptualization, data curation, investigation, and writing—original draft, review, and editing. GM: data curation and investigation. All authors contributed to the article and approved the submitted version.

## Footnotes

1
www.crd.york.ac.uk/PROSPERO/display_record.php?RecordID=284647

## References

[1] Reed GW Menon V. Reducing the incidence and mortality from myocardial infarction. *Lancet Public Health.* (2022) 7:e202–3. 10.1016/s2468-2667(22)00027-5 35247350

[2] Kapur NK Kanwar M Sinha SS Thayer KL Garan AR Hernandez-Montfort J Criteria for defining stages of cardiogenic shock severity. *J Am Coll Cardiol.* (2022) 80:185–98. 10.1016/j.jacc.2022.04.049 35835491

[3] Omer MA Tyler JM Henry TD Garberich R Sharkey SW Schmidt CW Clinical characteristics and outcomes of stemi patients with cardiogenic shock and cardiac arrest. *JACC Cardiovasc Interv.* (2020) 13:1211–9. 10.1016/j.jcin.2020.04.004 32438992

[4] Ibanez B James S Agewall S Antunes MJ Bucciarelli-Ducci C Bueno H 2017 ESC guidelines for the management of acute myocardial infarction in patients presenting with St-Segment elevation: the task force for the management of acute myocardial infarction in patients presenting with St-Segment elevation of the European Society of Cardiology (ESC). *Eur Heart J.* (2018) 39:119–77. 10.1093/eurheartj/ehx393 28886621

[5] Lo N Magnus Ohman E. Mechanical circulatory support in St-Elevation myocardial infarction. In: Watson TJ Ong PJL Tcheng JE editors. *Primary Angioplasty: A Practical Guide.* Singapore: Springer Copyright 2018, The Author(s) (2018). p. 253–73. 31314421

[6] Thiele H Ohman EM Desch S Eitel I de Waha S. Management of cardiogenic shock. *Eur Heart J.* (2015) 36:1223–30. 10.1093/eurheartj/ehv051 25732762

[7] Spiro J Doshi S. Use of left ventricular support devices during acute coronary syndrome and percutaneous coronary intervention. *Curr Cardiol Rep.* (2014) 16:544. 10.1007/s11886-014-0544-x 25326728

[8] Schmidt M Burrell A Roberts L Bailey M Sheldrake J Rycus PT Predicting survival after ecmo for refractory cardiogenic shock: the survival after veno-arterial-ecmo (save)-score. *Eur Heart J.* (2015) 36:2246–56. 10.1093/eurheartj/ehv194 26033984

[9] Muller G Flecher E Lebreton G Luyt CE Trouillet JL Brechot N The encourage mortality risk score and analysis of long-term outcomes after Va-Ecmo for acute myocardial infarction with cardiogenic shock. *Intensive Care Med.* (2016) 42:370–8. 10.1007/s00134-016-4223-9 26825953

[10] Choi KH Yang JH Park TK Lee JM Song YB Hahn JY Risk prediction model of in-hospital mortality in patients with myocardial infarction treated with venoarterial extracorporeal membrane oxygenation. *Rev Espanola Cardiol (English Ed).* (2019) 72:724–31. 10.1016/j.rec.2018.06.010 30037538

[11] Cheng R Hachamovitch R Kittleson M Patel J Arabia F Moriguchi J Complications of extracorporeal membrane oxygenation for treatment of cardiogenic shock and cardiac arrest: a meta-analysis of 1,866 adult patients. *Ann Thoracic Surg.* (2014) 97:610–6. 10.1016/j.athoracsur.2013.09.008 24210621

[12] Moher D Liberati A Tetzlaff J Altman DG Group P. Preferred reporting items for systematic reviews and meta-analyses: the prisma statement. *PLoS Med.* (2009) 6:e1000097. 10.1371/journal.pmed.1000097 19621072 PMC2707599

[13] Stang A. Critical evaluation of the newcastle-ottawa scale for the assessment of the quality of nonrandomized studies in meta-analyses. *Eur J Epidemiol.* (2010) 25:603–5. 10.1007/s10654-010-9491-z 20652370

[14] Chung SY Tong MS Sheu JJ Lee FY Sung PH Chen CJ Short-term and long-term prognostic outcomes of patients with St-Segment elevation myocardial infarction complicated by profound cardiogenic shock undergoing early extracorporeal membrane oxygenator-assisted primary percutaneous coronary intervention. *Int J Cardiol.* (2016) 223:412–7. 10.1016/j.ijcard.2016.08.068 27544596

[15] Lee WC Fang CY Chen HC Chen CJ Yang CH Hang CL Associations with 30-day survival following extracorporeal membrane oxygenation in patients with acute St Segment elevation myocardial infarction and profound cardiogenic shock. *Heart Lung J Crit Care.* (2016) 45:532–7. 10.1016/j.hrtlng.2016.08.006 27601212

[16] Semaan C Charbonnier A Pasco J Darwiche W Etienne CS Bailleul X Risk scores in St-Segment elevation myocardial infarction patients with refractory cardiogenic shock and veno-arterial extracorporeal membrane oxygenation. *J Clin Med.* (2021) 10:1–14. 10.3390/jcm10050956 33804450 PMC7957612

[17] Szczanowicz L Majunke N de Waha-Thiele S Tietz F Schürer S Kirsch K Predictors of clinical outcome after early veno-arterial extracorporeal membrane oxygenation in cardiogenic shock complicating St-Elevation myocardial infarction. *J Invasive Cardiol.* (2021) 33:E329–35. 33932279 10.25270/jic/20.00542

[18] Wu Y Zhang LT Feng L Huang XS Geng XB Li L. Effect of extra-corporealmembrane cxygenation combined with percutaneous coronary intervention oncardiac arrest patients due to acute myocardial infarction. *Chin Circ J.* (2018)33:561–6. 10.3969/j.issn.1000-3614.2018

[19] Li W Zhang JS Chen XF Mei Y Lv JR Hu DL Analysis of risk factors influencing the prognosis of the patients with acute St – Segment elevation myocardial infarction receiving venous – arterial extracorporeal membrane oxygenation. *Chin J Crit Care.* (2021) 41:635–9. 10.3969/j.issn.1002-1949.2021.07.022

[20] Cho S Lee W Lim SH Kang TS. Relationship between clinical outcomes and cardiopulmonary resuscitation time in patients with acute myocardial infarctiontreated by extracorporeal membrane oxygenation-assisted primary percutaneous coronary intervention. *Korean Circ J.* (2018) 48:705–15. 10.4070/kcj.2018.0121 30073808 PMC6072670

[21] Liu YW Li T Wang YY Duan DW Wang Y Liu BJ Analysis of the effectiveness of primary percutaneous coronary intervention assisted by extracorporeal membrane oxygenation in patients with critical acute myocardial infarction. *Chin J Intervent Cardiol.* (2015) 23:689–92.

[22] Liang QQ Wang BY Liu C. Analysis the influencing factors of clinical outcome of extracorporeal membrane oxygenation combined with percutaneous coronary intervention in rescue of cardiac arrest patients with acute myocardial infarction. *China Med.* (2021) 16:183–7.

[23] Sheu JJ Tsai TH Lee FY Fang HY Sun CK Leu S Early extracorporeal membrane oxygenator-assisted primary percutaneous coronary intervention improved 30-day clinical outcomes in patients with St-Segment elevation myocardial infarction complicated with profound cardiogenic shock. *Crit Care Med.* (2010) 38:1810–7. 10.1097/CCM.0b013e3181e8acf7 20543669

[24] Huang CC Hsu JC Wu YW Ke SR Huang JH Chiu KM Implementation of extracorporeal membrane oxygenation before primary percutaneous coronary intervention may improve the survival of patients with St-Segment elevation myocardial infarction and refractory cardiogenic shock. *Int J Cardiol.* (2018) 269:45–50. 10.1016/j.ijcard.2018.07.023 30077527

[25] Pahuja M Ranka S Chehab O Mishra T Akintoye E Adegbala O Incidence and clinical outcomes of bleeding complications and acute limb ischemia in stemi and cardiogenic shock. *Catheterization Cardiovasc Intervent.* (2020) 97:1129–38. 10.1002/ccd.29003 32473083 PMC8344361

[26] Fu HX Ma JF Hu MF Zhao ZN Wang Y Miao L. Outcome determinants in cardiac arrest patients secondary to acute myocardial infarction receiving extra-corporeal membrane oxygenation combined with percutaneous coronary intervention therapy. *Zhonghua Xin Xue Guan Bing Za Zhi.* (2017) 45:867–73. 10.3760/cma.j.issn.0253-3758.2017.10.011 29081177

[27] van den Brink FS Zivelonghi C Vossenberg TN Bleeker GB Winia VL Sjauw KD Va-Ecmo with IABP is associated with better outcome than Va-Ecmo alone in the treatment of cardiogenic shock in St-Elevation myocardial infarction. *J Invasive Cardiol.* (2021) 33:E387–92. 33893793 10.25270/jic/20.00085

[28] Brunner S Guenther SPW Lackermair K Peterss S Orban M Boulesteix A-L Extracorporeal life support in cardiogenic shock complicating acute myocardial infarction. *J Am College Cardiol.* (2019) 73:2355–7. 10.1016/j.jacc.2019.02.044 31072581

[29] Alonso-Fernandez-Gatta M Merchan-Gomez S Toranzo-Nieto I Gonzalez-Cebrian M Diego-Nieto A Barrio A Short-term mechanical circulatory support in elderly patients. *Artif Organs.* (2021) 46:867–77. 10.1111/aor.14117 34780090

[30] Blutinger AL Zollo AM Weltman J Prittie J. Prospective evaluation of plasma lactate parameters for prognosticating dogs with shock. *J Vet Emerg Crit Care (San Antonio).* (2021) 31:351–9. 10.1111/vec.13046 33709568

[31] Scolari FL Schneider D Fogazzi DV Gus M Rover MM Bonatto MG Association between serum lactate levels and mortality in patients with cardiogenic shock receiving mechanical circulatory support: a multicenter retrospective cohort study. *BMC Cardiovasc Disord.* (2020) 20:496. 10.1186/s12872-020-01785-7 33234107 PMC7687839

[32] Flegal K Kit B Orpana H Graubard B. Association of all-cause mortality with overweight and obesity using standard body mass index categories: a systematic review and meta-analysis. *JAMA.* (2013) 309:71–82. 10.1001/jama.2012.113905 23280227 PMC4855514

[33] Ortega FB Lavie CJ Blair SN. Obesity and cardiovascular disease. *Circ Res.* (2016) 118:1752–70. 10.1161/circresaha.115.306883 27230640

[34] Oliveros H Villamor E. Obesity and mortality in critically ill adults: a systematic review and meta-analysis. *Obesity (Silver Spring, Md).* (2008) 16:515–21. 10.1038/oby.2007.102 18239602

[35] Salna M Fried J Kaku Y Brodie D Sayer G Uriel N Obesity is not a contraindication to veno-arterial extracorporeal life support. *Eur J Cardiothorac Surg.* (2021) 60:831–8. 10.1093/ejcts/ezab165 33969398

[36] Sreenivasan J Khan MS Sharedalal P Hooda U Fudim M Demmer RT Obesity and outcomes following cardiogenic shock requiring acute mechanical circulatory support. *Circ Heart Fail.* (2021) 14:e007937. 10.1161/CIRCHEARTFAILURE.120.007937 33706552

[37] Park S Yang J Park T Cho Y Sung K Chung C Developing a risk prediction model for survival to discharge in cardiac arrest patients who undergo extracorporeal membrane oxygenation. *Int J Cardiol.* (2014) 177:1031–5. 10.1016/j.ijcard.2014.09.124 25443259

[38] Singer B Reynolds JC Lockey DJ O’Brien B. Pre-hospital extra-corporeal cardiopulmonary resuscitation. *Scand J Trauma Resuscitation Emerg Med.* (2018) 26:21. Epub 10.1186/s13049-018-0489-y 29587810 PMC5870373

[39] Park JH Song KJ Shin SD Ro YS Hong KJ. Time from arrest to extracorporeal cardiopulmonary resuscitation and survival after out-of-hospital cardiac arrest. *Emerg Med Australas.* (2019) 31:1073–81. 10.1111/1742-6723.13326 31155852

[40] Lee H-H Kim HC Ahn C-M Lee S-J Hong S-J Yang JH Association between timing of extracorporeal membrane oxygenation and clinical outcomes in refractory cardiogenic shock. *JACC Cardiovasc Intervent.* (2021) 14:1109–19. 10.1016/j.jcin.2021.03.048 34016408

[41] Rathore SS Curtis JP Chen J Wang Y Nallamothu BK Epstein AJ Association of door-to-balloon time and mortality in patients admitted to hospital with St Elevation myocardial infarction: national cohort study. *BMJ (Clin Res ed).* (2009) 338:b1807. 10.1136/bmj.b1807 19454739 PMC2684578

[42] Parlow S Di Santo P Mathew R Jung RG Simard T Gillmore T The association between mean arterial pressure and outcomes in patients with cardiogenic shock: insights from the Doremi trial. *Eur Heart J Acute Cardiovasc Care.* (2021) 10:712–20. 10.1093/ehjacc/zuab052 34382063

[43] Olson SR Murphree CR Zonies D Meyer AD Mccarty OJT Deloughery TG Thrombosis and bleeding in extracorporeal membrane oxygenation (Ecmo) without anticoagulation: a systematic review. *ASAIO J.* (2021) 67:290–6. 10.1097/mat.0000000000001230 33627603 PMC8623470

[44] Laimoud M Ahmed W. Acute neurological complications in adult patients with cardiogenic shock on veno-arterial extracorporeal membrane oxygenation support. *Egyptian Heart J (EHJ) Off Bull Egyptian Soc Cardiol.* (2020) 72:26. 10.1186/s43044-020-00053-5 32449052 PMC7246233

[45] Lamb KM DiMuzio PJ Johnson A Batista P Moudgill N McCullough M Arterial protocol including prophylactic distal perfusion catheter decreases limb ischemia complications in patients undergoing extracorporeal membrane oxygenation. *J Vasc Surg.* (2017) 65:1074–9. 10.1016/j.jvs.2016.10.059 28342510

[46] Juo YY Skancke M Sanaiha Y Mantha A Jimenez JC Benharash P. Efficacy of distal perfusion cannulae in preventing limb ischemia during extracorporeal membrane oxygenation: a systematic review and meta-analysis. *Artif Organs.* (2017) 41:E263–73. 10.1111/aor.12942 28762511

[47] Gu M Mei XL Zhao YN. A review on extracorporeal membrane oxygenation and kidney injury. *J Biochem Mol Toxicol.* (2021) 35:e22679. 10.1002/jbt.22679 33325616

