# Supplementary material for: Effects and safety of extracorporeal membrane oxygenation in the treatment of patients with ST-segment elevation myocardial infarction and cardiogenic shock: A systematic review and meta-analysis
Source: Front Cardiovasc Med. 2022 Sep 27;9:963002. doi: 10.3389/fcvm.2022.963002 (PMC9552800; doi:10.3389/fcvm.2022.963002)

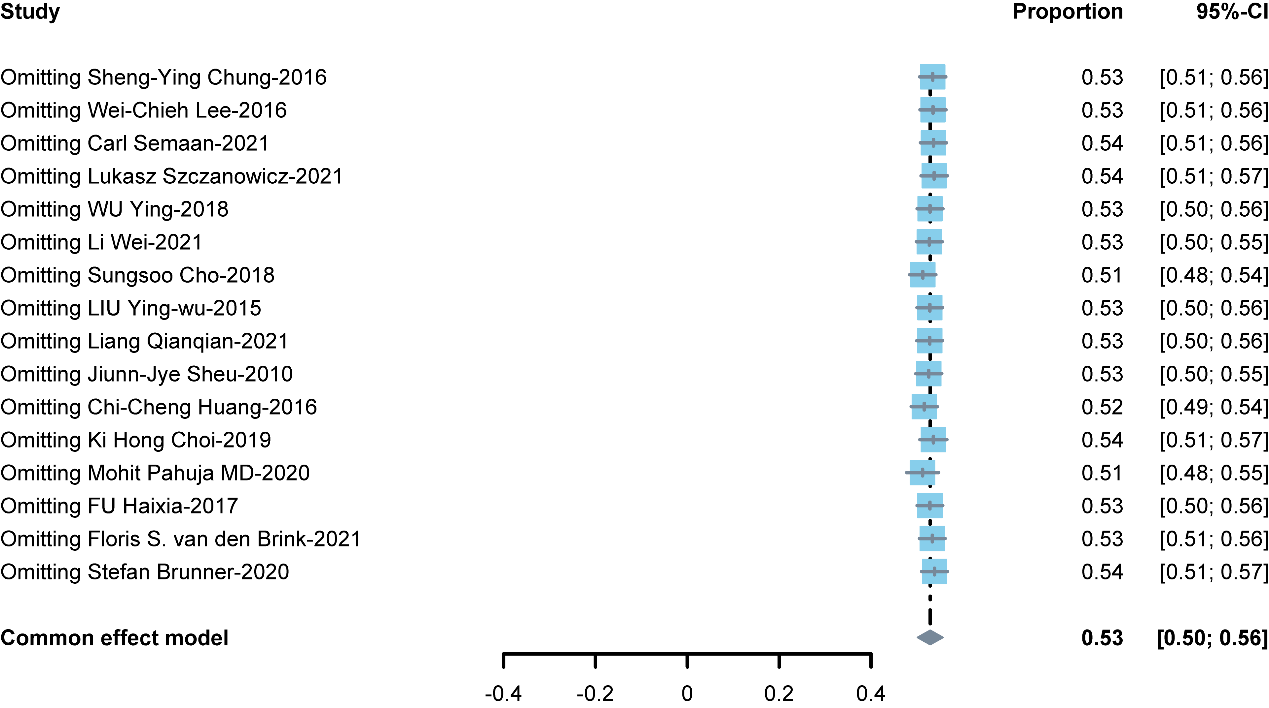


Supplemental Figure 1. Sensitivity analysis graph of short-term mortality for patients on VA-ECMO for CS complicating STEMI





Supplemental Figure 2. Forest plots of the meta-analysis of age


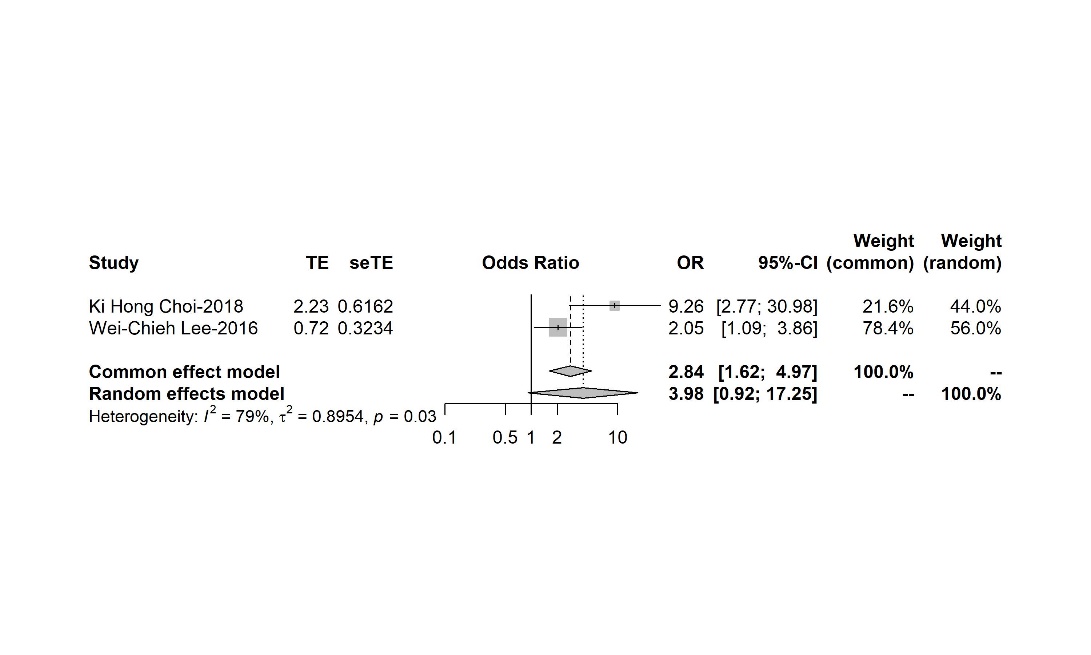


Supplemental Figure 3. Forest plots of the meta-analysis of BMI


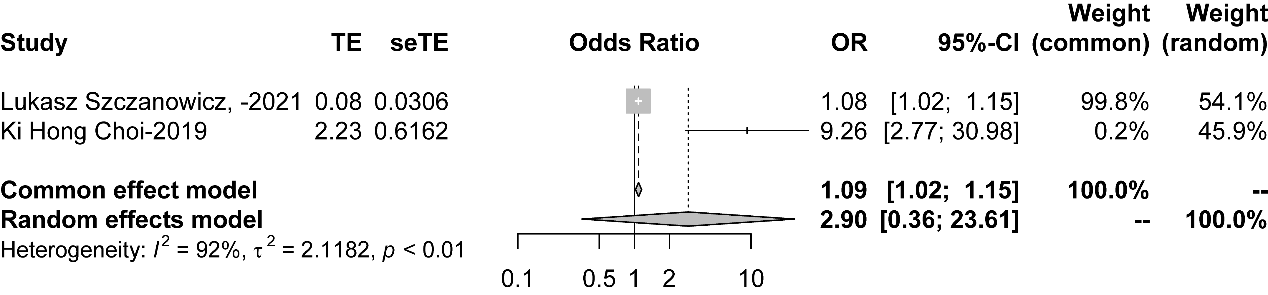


Supplemental Figure 4. Forest plots of the meta-analysis of lactate


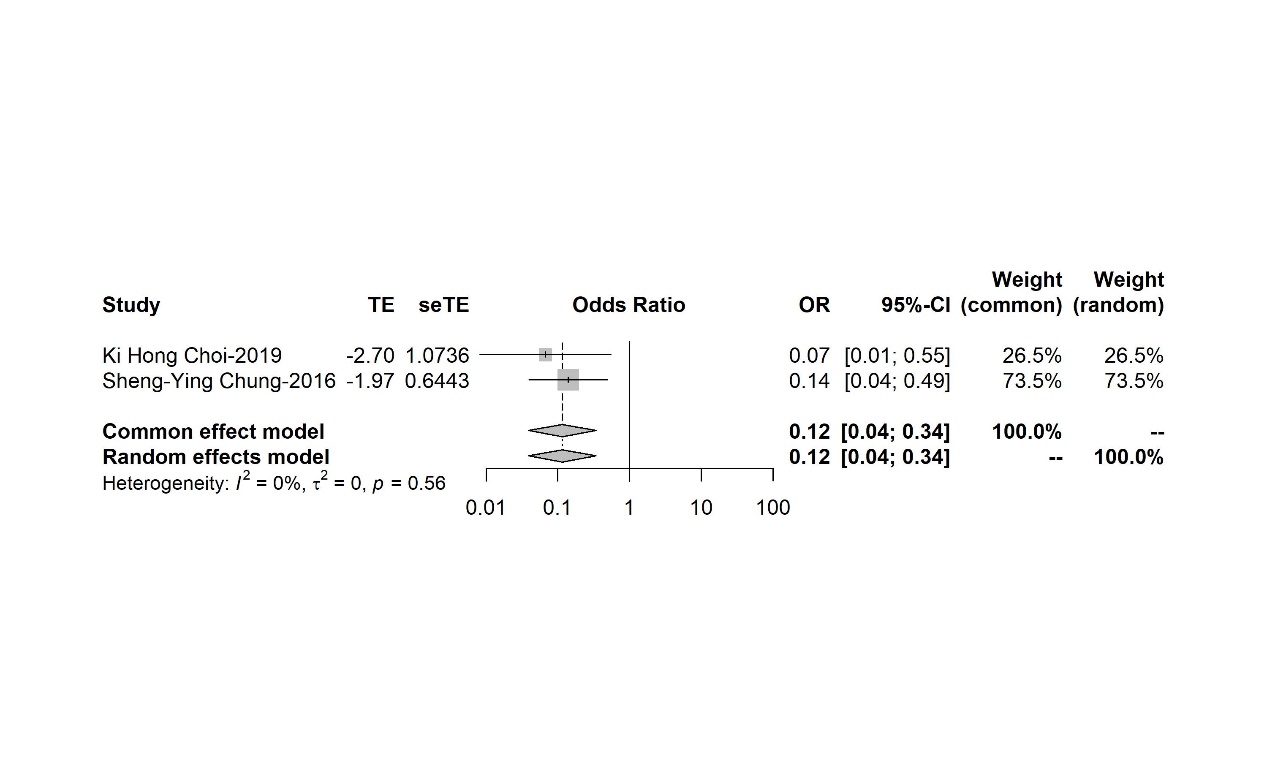


Supplemental Figure 5. Forest plots of the meta-analysis of TIMI-3 flow after PCI





Supplemental Figure 6. Forest plots of the meta-analysis of anterior wall infarction


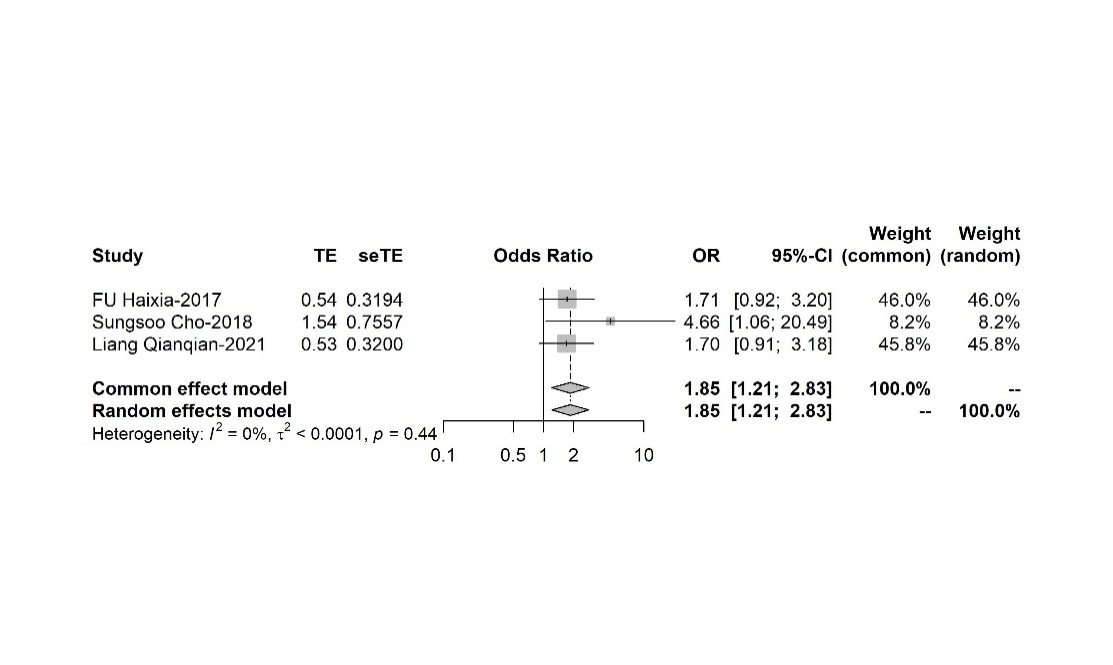


Supplemental Figure 7. Forest plots of the meta-analysis of CPR time


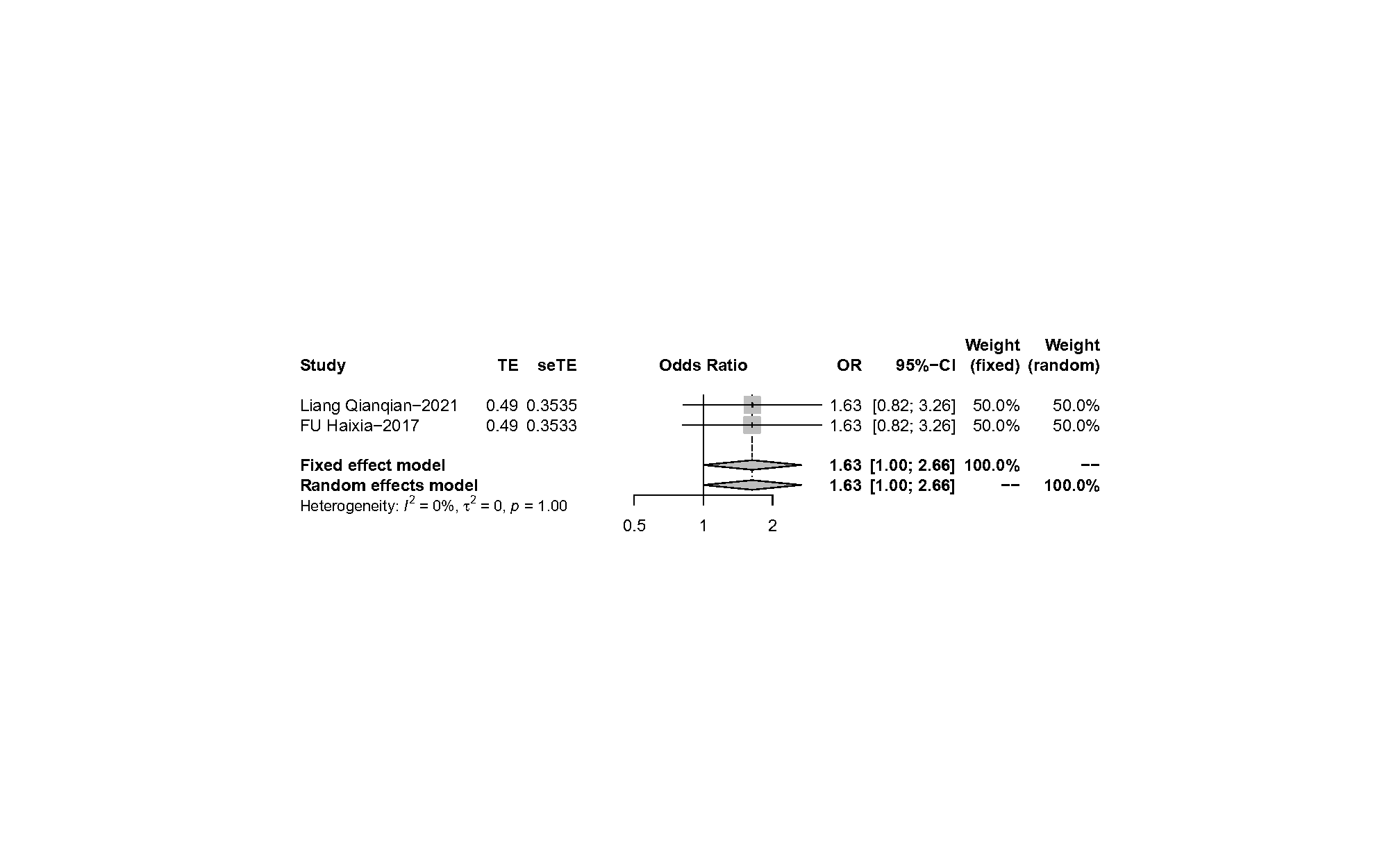


Supplemental Figure 8. Forest plots of the meta-analysis of arrest to ECMO time

Appendix I

1.Embase


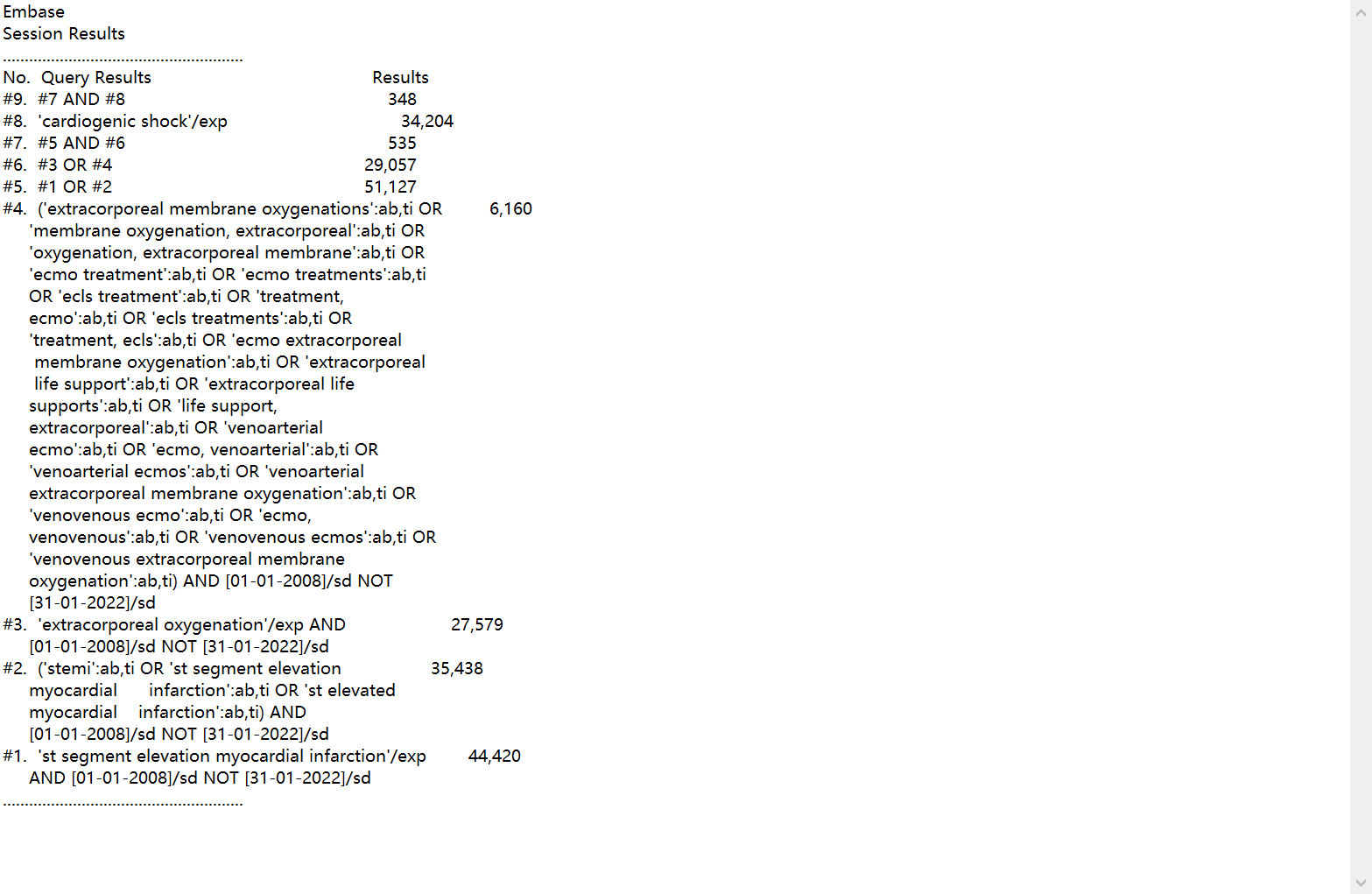


2.PUBMED


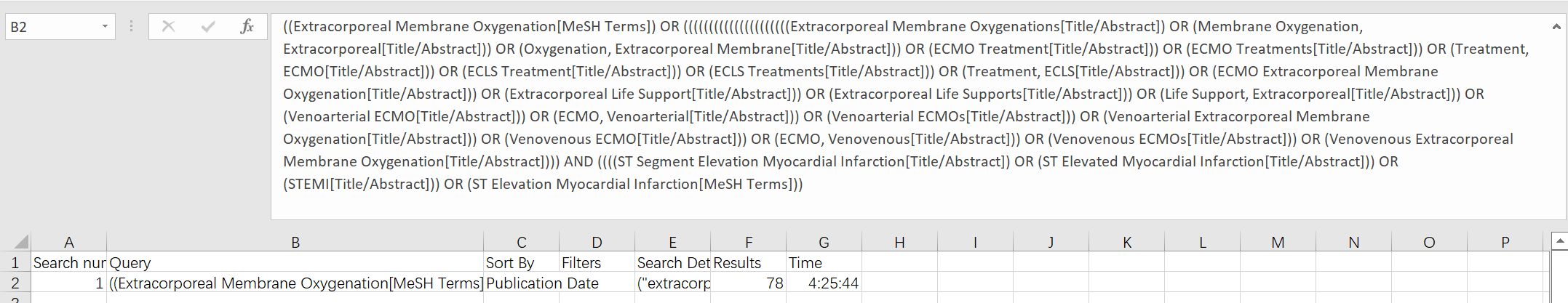


3.Web of science


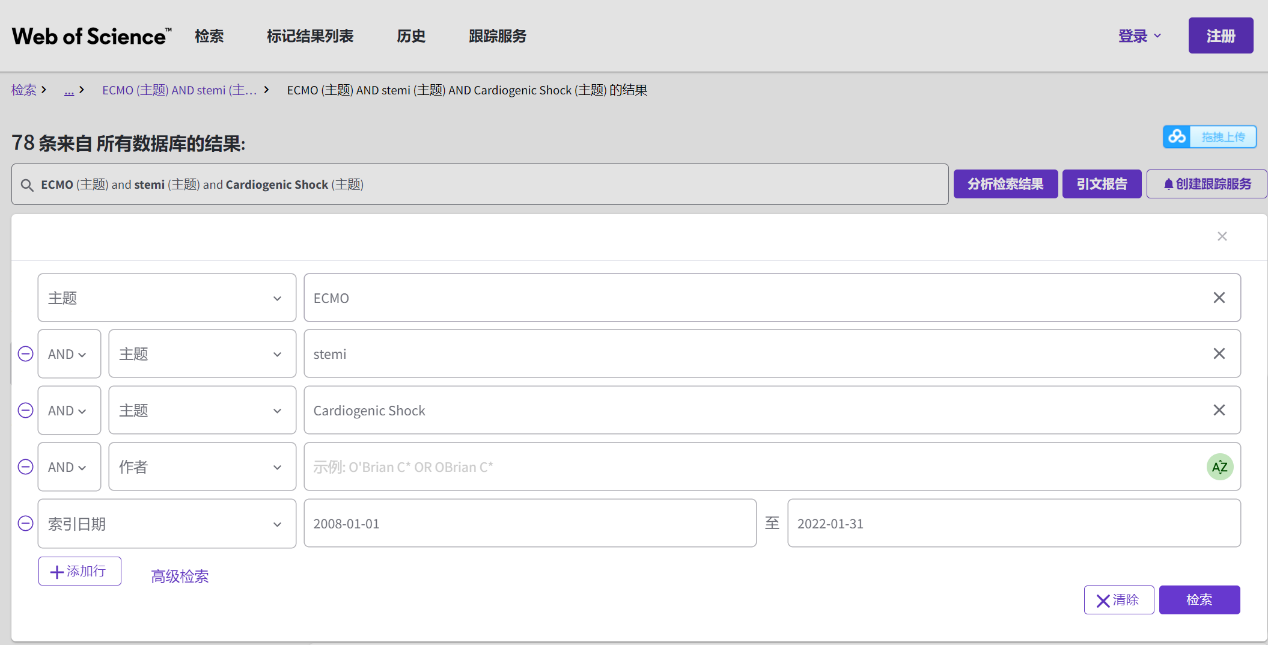


4. Cochrane


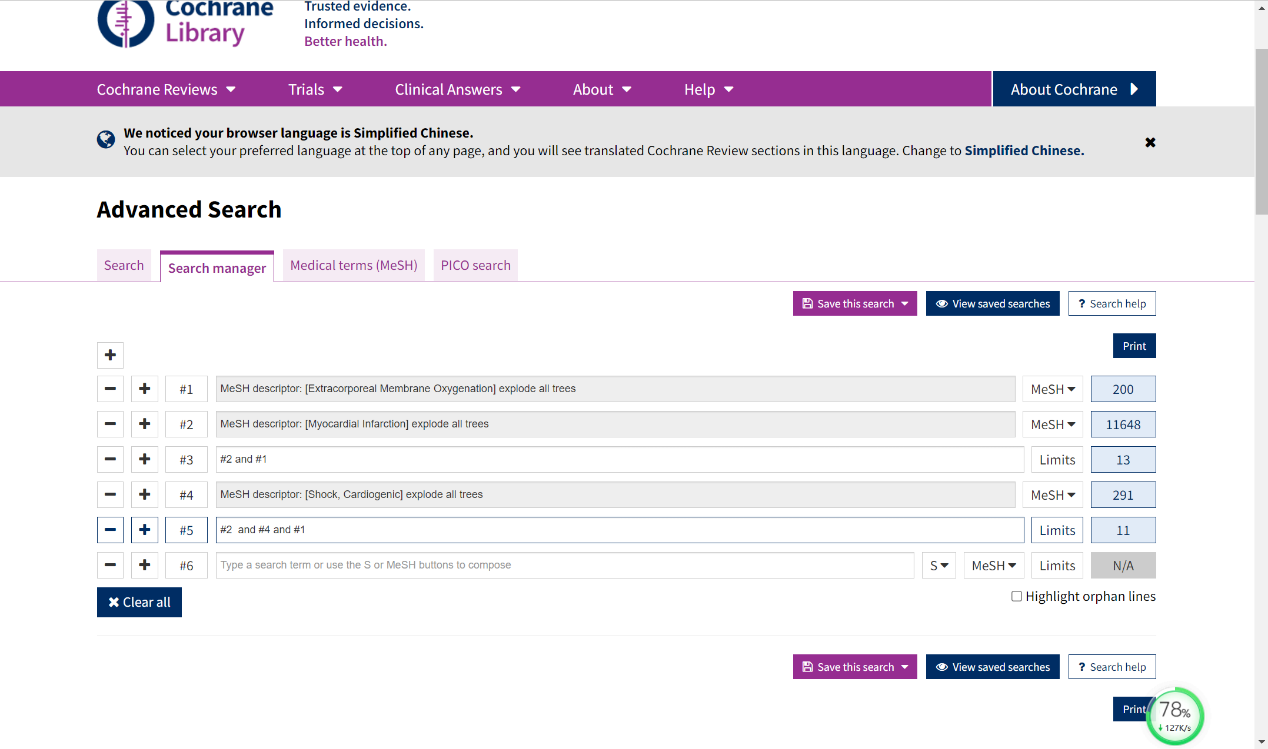


5. China National Knowledge Infrastructure (CNKI)


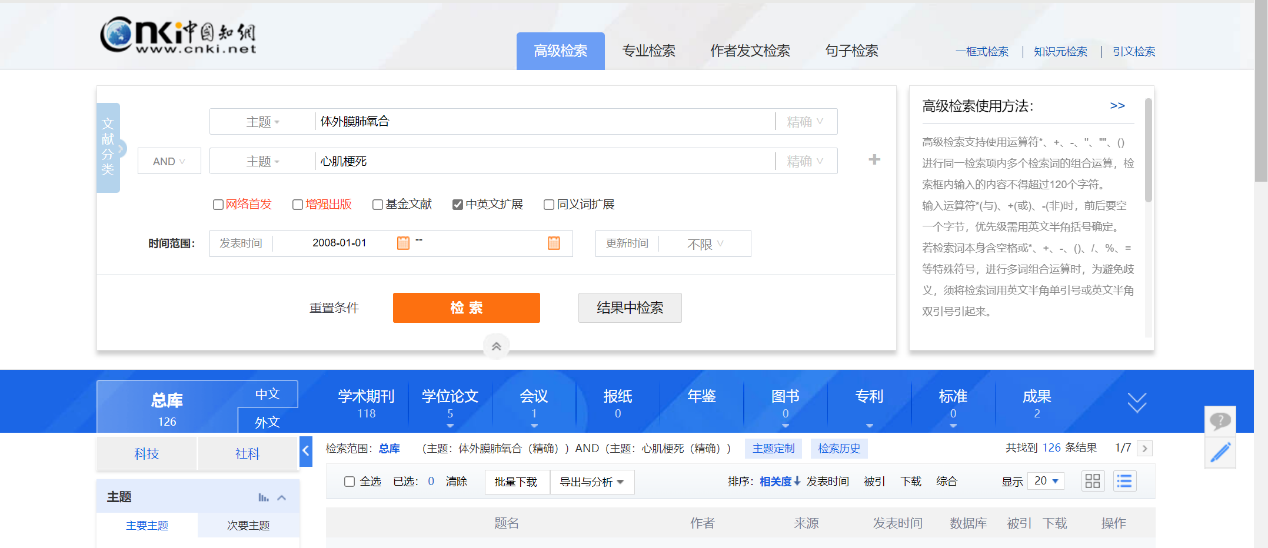


6. Wanfang Database


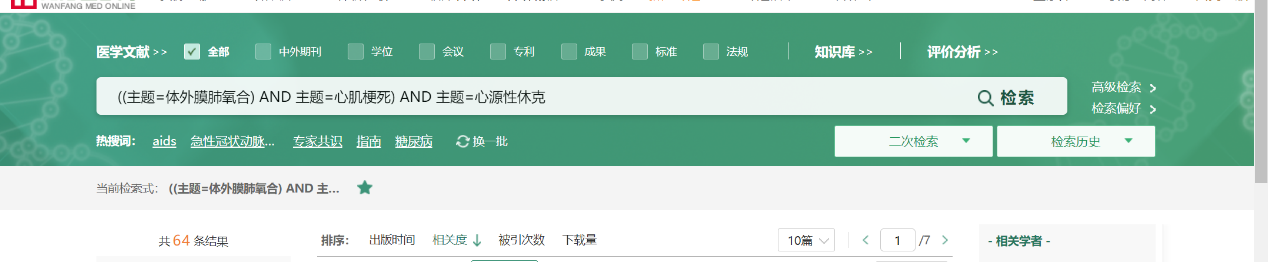

Supplement: Supplementary file 1 [file Data_Sheet_1.docx]
